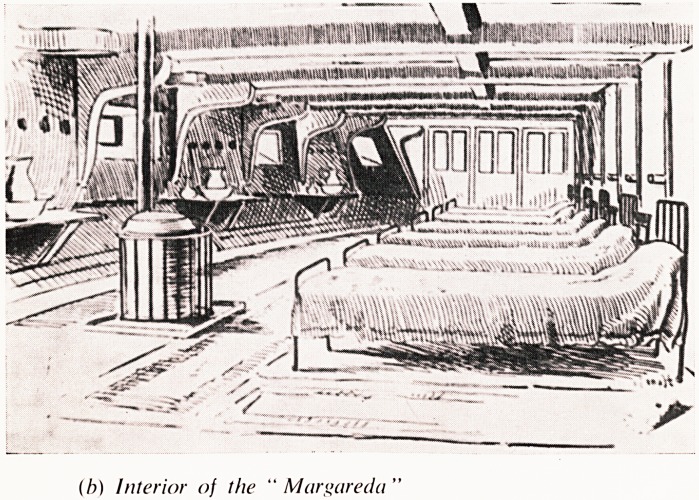# A Short History of Isolation Accommodation in Bristol

**Published:** 1968-10

**Authors:** D. W. Wright


### A SHORT HISTORY OF ISOLATION ACCOMMODATION IN BRISTOL

BY

D. W. WRIGHT

Dr. J. F. J. Sykes, in his interesting " Public Health Problems ", pointed out that in the ancient world, flight was the only resort in the presence of epidemic disease, and that to this fact may be ascribed the depopulation, ruin, and disappearance of many cities of antiquity. Approaching more modern times, the spirit of combating rather than fleeling from infection appeared, and it assumed the form of isolation. Isolation may either be by exclusion or by seclusion. At first it was practised by wholesale methods in the old " quarantine " and " cordon " systems; in more recent years these were displaced by the methods of exclusion and seclusion in detail, that is, by their application to individual cases. Nowadays strict isolation is necessary for only a limited number of infectious diseases, one example of which is smallpox. It is perhaps a sobering thought that in the 18th century an infected person escaping from quarantine was liable to death as a felon.

The provision of isolation hospital accommodation in Bristol can be traced back at least as far as the fourteenth century when three leper hospitals existed in Bristol, pointing perhaps to an extreme local prevalence of the disease as well as testifying to the general endemicity of this disease. There are accounts in the literature about St. Peter's Hospital, St. Peter's Street, Bristol, being swept by cholera in the 19th century, and in the preceding century (in 1799) Dr. Beddoe's pneumatic institute in Dowry Square (off Hotwell Road, where Davy discovered the properties of " laughing gas ") had to be closed to cope with an outbreak of typhus fever.

It would seem that we were rather slow to provide proper isolation hospitals. The lesson was partly learned in regard to one class of communicable disease; the English cholera epidemics of 1832, 1847, and 1854 had directed attention to the need, and the action taken on this impulse undoubtedly saved many lives.

Cholera and its congener, enteric fever, indirectly did an immensity of good in emphasising the need for good sanitary conditions in preventing a limited class of communicable disease. But as in combating the spread of such diseases hospitals play a secondary and a comparatively unimportant part, the very prominence of these diseases in initiating modern preventive medicine may have tended to obscure the importance of isolation in the large class of diseases which spread more immediately from person to person, for the repression of which, therefore, efficient hospital isolation was a first necessity.

Whatever was the full cause, or causes, it is certain that the growth of the Isolation Hospital provision was slow, and that the transition from rough and ready provision to properly equipped and properly administered Isolation Hospitals (following the lead of the General Hospitals and Infirmaries) was slower.

In the early years of sanitary effort in Bristol, hospital provision for communicable diseases was associated with the Guardians of the Poor who were the only authorities dealing with such matters. Thus in 1849 the nursing of cholera patients, and later (in 1865) the isolation of typhus, was undertaken by the three Boards of Guardians then having jurisdiction within the City.

In 1886 the care of the infectious sick was still shared by the Sanitary Authority with the Guardians. The only hospitals then belonging to the Corporation of Bristol were two temporary wooden buildings, for 12 patients each, situated close together in a yard in St. Philip's Marsh, near the Feeder Canal beyond Temple Meads Station, and thus unsuitable for isolating more than one kind of disease, although nominally intended for fevers and smallpox. The prevalence of smallpox in 1887-88 (over 300 cases occurred in the city) and the concurrent prevalence of scarlet fever, emphasised the need for more adequate hospital accommodation; and the additional rise in the number of cases of diphtheria only served further to emphasise this need. The increase in the number of cases of smallpox in the city iin 1887-88 occurred during the period of the Gloucester smallpox epidemic of 1886-1895 when, out of 15,682 children born during that period, 3,176 died of smallpox.

The Notification Act of 1889 first gave full information as to the prevalence of communicable disease.

On March 25th, 1892, the Sanitary Authority purchased a site at Novers Hill, Knowle. It covered 13 acres and was reserved solely for the treatment of smallpox cases and could accommodate approximately 50 patients. In 1893-94, because of a further smallpox epidemic, additional isolation facilities were pressed into service for the city and these included the Port Hospital ship (The Margareda) moored at the mouth of the River Avon, and the Avonmouth Port Shire Hospital, providing respectively 25 and 16 beds. The ship went out of use in 1916. With the sites at Novers Hill, St. Philip's Marsh, and Clift House, this gave nearly 300 beds available; but more permanent accommodation was considered essential, worked out on the basis of 1 bed per 1,000 population.

In 1894 the then Medical Officer of Health, Dr. D. S. Davies, reported that (after eight years of discussion and negotiation) a site had been purchased at Ham Green. This was bought by the Bristol Corporation in September, 1894, from Sir George Edwards for the same sum as he had paid for it the previous year, namely £8,695—this for an estate of approximately 100 acres, a mansion, and stables, situated 4½ miles from the city with access by road and river. In the first instance it was to have 76 beds, the estimated cost of which was £27,592, the cost per bed being £363, compared with approximately £10,000 per bed today. The Hospital was officially opened by the then Lord Mayor of Bristol (The Right Hon. Councillor Herbert Ashman) and, with its initial 76 beds, was served by both road and river, patients somethimes coming by boat to a landing-stage situated near the mansion house and known as the " Adam and Eve ".

For the first two years, the Hospital itself (comprising 38 acres) was used only for typhoid cases. It was constructed upon the pavilion principle, enunciated by Tenon in 1787-88, and first applied in the construction of the Lariboisiere Hospital in Paris in 1854, since which date numerous modifications and improvements had led to its general adoption, especially for permanent Isolation Hospitals to which the principle was especially suited.

*St. Peter's Hospital*

(*a*) *The Port Hospital ship "Margareda" in the Avon*

(*b*) *Interior of the "Margareda"*

Each pavilion or block was separated from the others, or connected only by a covered way. Independent provision was made in each pavilion for both sexes and for the necessary nursing, store room, bathroom, water closet, and other accommodation.

The changing pattern of infectious disease brought about by natural causes, advances in preventive medicine, and new treatments, can perhaps be best illustrated, with relation to the City of Bristol, by a few examples.

In 1929 there were 1,047 cases of diphtheria with 45 deaths. This year an epidemic of virulent cases taxed the accommodation of the Hospital in the autumn. About this time, immunisation on a larger scale than hitherto was started in the area. In 1930, 5,485 persons had been inoculated since the beginning of the campaign. This was about a 50% response from the public to the appeal to have their children Schick-tested and immunised if necessary. It was not, however, until 1941-42, after the Jameson Radio Appeal, that mass immunisation can really be said to have started.

In 1950 no cases of diphtheria were reported in the city (the first year in which there had been no new cases); and now in 1968, there have been no new cases for nearly eighteen years.

The incidence of scarlet fever used to be high; between 1901-1905 there were 9,441 cases with 226 deaths, and in 1928 there were 1,211 cases with 3 deaths. Its incidence is now very small and scarlet fever is no longer a problem.

During the 1953 poliomyelitis epidemic about 200 patients were treated in Ham Green Hospital, but there has been no new case in the Hospital for several years.

In 1950 there were nearly 500 beds available to the city for the care of tuberculous patients in the hospitals or sanatoria at Ham Green, Charterhouse, Southmead, Frenchay Sanatorium, Frenchay Hospital, and Winsley. In 1966 only 76 new cases of pulmonary tuberculosis were notified in the city, and today less than 30 beds (at Ham Green Hospital) are required for tuberculous patients.

As already mentioned advances in the prevention and treatment of many of the infectious diseases have made strict isolation no longer necessary except for a limited number of these diseases. Smallpox is a notable exception because of its infectivity, its possible serious effects, and the relative lack of effective treatment.

Ham Green is now mainly an " acute " hospital, and many of its 500 beds are used for patients having other than infectious diseases, though some beds are always kept immediately available for the care of smallpox patients should the need arise.

SOURCES

1. Sykes, J. F. J. Public Health Problems. Contemporary Science Series. London, Walter Scott. 1892.

2. Bond, Francis T. " The Story of the Gloucester Epidemic Smallpox ". April issue of the Public Health 1897 by Dr. Campbell, M.O.H. Gloucester.

ACKNOWLEDGEMENTS :

Some of the material for this article came from a copy of the programme issued for the Opening Ceremony of the Hospital on July 12th, 1899, which is available in the Hospital, and from copies of the published Annual Reports of the Medical Officer of Health, Bristol.
I am indebted to Dr. R. C. Wofinden, the present Medical Officer of Health, for some additional information and helpful advice, and for the photographs.

## Figures and Tables

**Figure f1:**
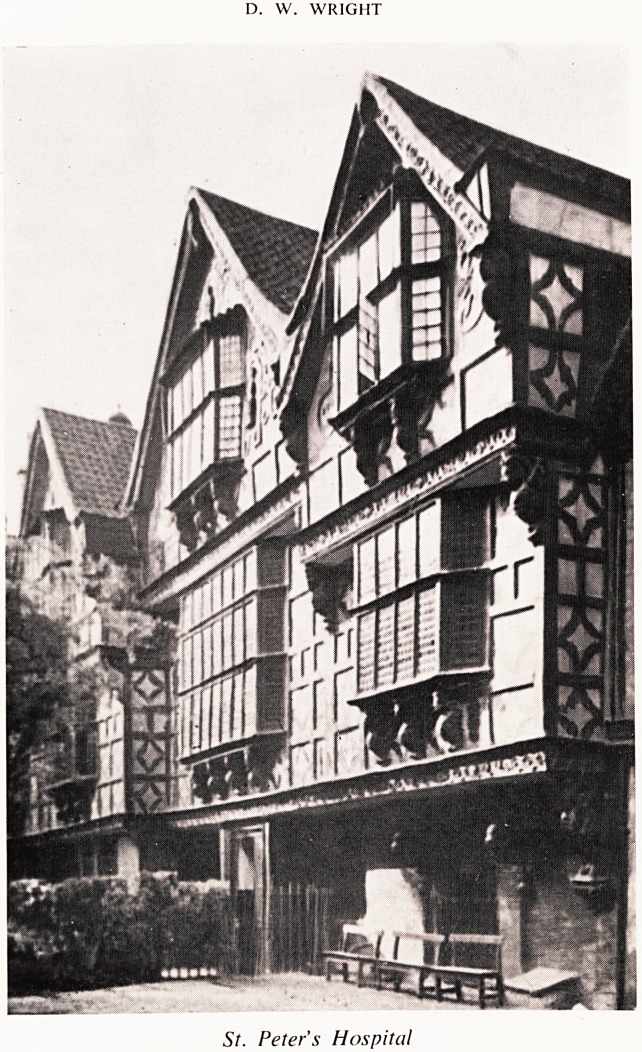


**Figure f2:**
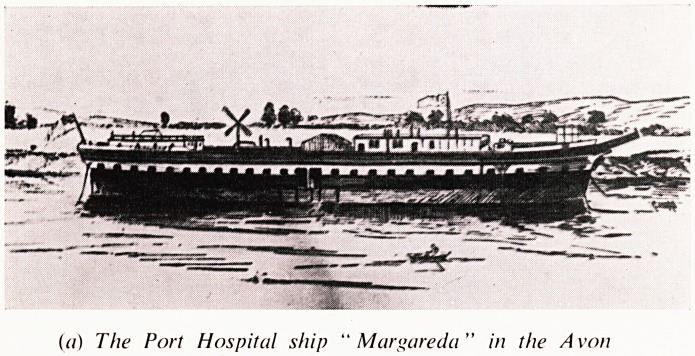


**Figure f3:**